# Diagnostic Utility and Economic Impact of a Sequential MRI Protocol in Axial Spondyloarthritis: A Retrospective Cohort Study

**DOI:** 10.7759/cureus.99688

**Published:** 2025-12-20

**Authors:** Jack Shi Jie Yuan-Doré, Sateesh Kumar, Luke Kostanjsek, Syed-Ali Tahir, Sian Bamford, William Tilden, Hasan Tahir

**Affiliations:** 1 Department of Rheumatology, Royal Free London NHS Foundation Trust, London, GBR; 2 Department of Geriatrics, Barnet Hospital, London, GBR; 3 Department of Acute Medicine, Royal Free London NHS Foundation Trust, London, GBR; 4 School of Medicine, Imperial College London, London, GBR; 5 Department of Physiotherapy, Royal Free London NHS Foundation Trust, London, GBR; 6 Department of Radiology, Royal Free London NHS Foundation Trust, London, GBR; 7 Division of Medicine, University College London, London, GBR

**Keywords:** : ankylosing spondylitis, assessment of spondyloarthritis international society (asas), axial spondyloarthritis, health economic evaluation, muskuloskeletal mri, non-radiographic axial spondyloarthritis

## Abstract

Background

Diagnosis of axial spondyloarthritis (axSpA) is a diagnosis based on both clinical history and characteristic radiographic and MRI findings. MRI is increasingly used to diagnose non-radiographic axSpA. The main question is whether all patients need both MRI of the sacroiliac joint (SIJ) and the spine to achieve a diagnosis of axSpA. Here, we propose a sequential MRI SIJ followed by MRI spine only for those patients who do not have inflammatory lesions on MRI SIJ, to optimise MRI use and save resources.

Methods

This is an observational retrospective cohort study conducted in our tertiary centre specialist axSpA clinic. We included only patients with a diagnosis of axSpA with both MRI spine and SIJ, who were referred between January 2024 and November 2024 to our tertiary service. Radiology reports were then reviewed by clinicians and determined if the MRI SIJ imaging alone was sufficient for the diagnosis, or if the MRI whole spine imaging was necessary to achieve a diagnosis of axSpA. Cost minimization analysis was performed. Logistical regression was performed to identify potential clinical characteristics that may predict the need for both SIJ and spine MRI for diagnosis.

Results

A total of 62 patients were included in this analysis. Of these, 51 patients (82.3%) had changes on MRI SIJ alone that were diagnostic for active inflammatory lesions compatible with a diagnosis of axSpA, and only 11 patients (17.7%) had inflammatory lesions in the spine, hence would have needed further spinal MRI to identify inflammatory lesions in addition to SIJ MRI. A cost minimisation analysis of utilising a sequential scanning protocol, where patients would initially have an MRI SIJ that would be reviewed by a clinician to determine whether further MRI spine was needed, identified potential cost savings of £11,118 and 34.0 hours of active scanning time. Logistical regression identified that there was a trend that patients with inflammatory bowel disease (IBD) had a greater odds ratio of 3.80 (95%CI 0.537- 26.952, p = 0.181) of needing a spinal MRI in addition to SIJ MRI to achieve an axSpA diagnosis. A history of human leukocyte antigen (HLA)-B27 positivity, psoriasis, and uveitis was not associated with a greater likelihood of needing an MRI spine in addition to MRI SIJs to achieve axSpA diagnosis.

Conclusion

Our analysis has demonstrated that there are potential significant resource savings from a cost and MRI-scanning time perspective by the implementation of a sequential MRI scanning protocol in patients with a high clinical suspicion of axSpA. Additionally, we have identified IBD as a clinical variable that may potentially predict the need for further MRI spine imaging in addition to MRI SIJ imaging.

## Introduction

Axial spondyloarthritis (axSpA) is a chronic inflammatory condition that predominantly affects the sacroiliac joints (SIJs) and spine, presenting as persistent back pain and stiffness. Diagnosis is made based on clinical assessment and supportive imaging findings. To facilitate earlier recognition, the Assessment of SpondyloArthritis International Society (ASAS) developed classification criteria in 2009 for axial spondyloarthritis, intended primarily for research settings [1,2]. These criteria require patients to have chronic back pain for ≥ 3 months with onset before age 45. From there, entry can proceed via two arms: the imaging arm (presence of sacroiliitis on radiographs or MRI plus ≥ 1 clinical feature of spondyloarthritis) or the clinical arm (human leukocyte antigen (HLA)‑B27 positivity plus ≥ 2 additional clinical features) [1].

These criteria highlight the role of imaging in axSpA. Given that radiographic changes on X-rays can take years to develop, there is emphasis on magnetic resonance imaging (MRI) in clinical practice to facilitate earlier diagnosis of axSpA. The ASAS MRI working group has developed standardised definitions of axSpA lesions in both the SIJs and spine [3-6]. A positive MRI requires evidence of inflammatory lesions. For the sacroiliitis, this would be bone marrow oedema (BMO) in the typical subchondral region of the SIJs, and for vertebral inflammatory lesions, this would be corner, endplate, or facet joint lesions. It is important to note that structural lesions may support interpretation but are not sufficient on their own.

It is well-documented that axSpA-positive MRI findings are identified both in the whole vertebral column and SIJs [7-9]. Current UK guidance from the British Society for Spondyloarthritis (BRITSpA) recommends MRI imaging of both SIJs with the whole spine in a unified protocol [10]. This is due to the presence of evidence suggestive that there are subsets of axSpA patients who develop only spinal lesions without sacroiliitis [9]. This differs from the European Alliance of Associations for Rheumatology (EULAR) recommendations, where they recommend against MRI spine imaging, but it is important to note that this recommendation is older and precedes the ASAS MRI working group’s definitions of vertebral inflammatory lesions [11]. Given the variable availability of MRI across different institutions, it is pertinent to consider whether it is necessary to image both MRI SIJs and the whole spine in patients who have positive findings on MRI SIJs alone.

Our retrospective cohort study aims to answer this question. Our aim is to identify the proportion of patients who had diagnostic imaging findings on MRI SIJs alone and those patients who needed both spinal and SIJs MRI. We reviewed the MRI reports of patients who have a diagnosis of axSpA who had both spine and SIJ MRI during their diagnostic work-up, separating those with positive MRI findings based on MRI SIJ imaging alone and those who had positive MRI findings on spinal imaging. Our secondary objective was also to perform a cost minimisation analysis of using a sequential MRI protocol, where patients would only have spinal MRI should MRI SIJs not show diagnostic imaging findings of axSpA, in comparison to imaging all patients with both MRI SIJ and spine.

## Materials and methods

Study design and setting

This was an observational retrospective cohort study conducted in our tertiary centre specialist axial spondyloarthritis clinic at Barnet and Edgeware Hospitals within the Royal Free NHS Foundation Trust, London, United Kingdom. This specialist clinic receives referrals from primary care for patients in whom there is a clinical suspicion of axSpA, with specific referral criteria. Local referral criteria require patients to meet four out of the five criteria: age of onset of back pain less than 45 years of age, back pain that is gradual, improves with exercise, does not improve with rest, and pain at night, which improves on getting up.

Eligibility criteria and sample size

Our inclusion criteria were as follows: patients over the age of 18 with a diagnosis of axSpA, who were referred between January 2024 and November 2024 to our tertiary axSpA service, were biologic-naïve at the time of imaging, and had both MRI whole spine and MRI SIJ imaging performed between January 2023 and November 2024, with written reports available for review. Exclusion criteria included patients who did not have both spinal and SIJ MRI imaging.

A total of 62 patients met the eligibility criteria and were included in this analysis.

MRI of spine and SIJ

All patients had MRI sequences that included sagittal short Tau inversion recovery (STIR)/T1 spine and coronal oblique SIJ. Local spinal MRI protocols for the investigation of axSpA involve abbreviated imaging, which includes the cervicothoracic and lumbosacral spine but without axial imaging. A minority of patients obtained MRIs at other diagnostic imaging centres. These images were imported and re-reviewed by two local experienced musculoskeletal radiologists with a specialist interest in axSpA imaging and a rheumatologist at our axSpA imaging multidisciplinary team meeting.

Data collection

Clinical and demographic data were collected via review of electronic patient records by investigators. These data included age, sex, weight, HLA-B27 status, and previous concomitant diagnoses of uveitis, psoriasis, or inflammatory bowel disease (IBD). Radiology reports were then reviewed by two clinicians who were unblinded to clinical information against the definitions as defined by the ASAS MRI working group [3,5], such as vertebral corner, endplate, and facet inflammatory lesions and bone marrow oedema around the SIJ. Based on the radiology report, it was determined whether the MRI SIJ imaging alone was sufficient for the diagnosis or if MRI whole spine imaging was necessary to achieve a diagnosis of axSpA.

Statistical analysis

The primary endpoint of the statistical analysis was the proportion of patients who required an MRI of the whole spine in order to make the diagnosis of axSpA. A 95% confidence interval (CI) was calculated for this proportion using the Wilson interval. Following this, a posthoc exploratory analysis for characteristics that may be predictive of the need for MRI whole spine was performed. A logistic regression model was created to estimate the likelihood of an MRI whole spine being required, using demographic and clinical variables.

The data for this study were extracted, prepared, and analysed using RStudio version 2025.09.0 (R Foundation for Statistical Computing, Vienna, Austria) [12].

Cost minimisation analysis

**Figure 1:**
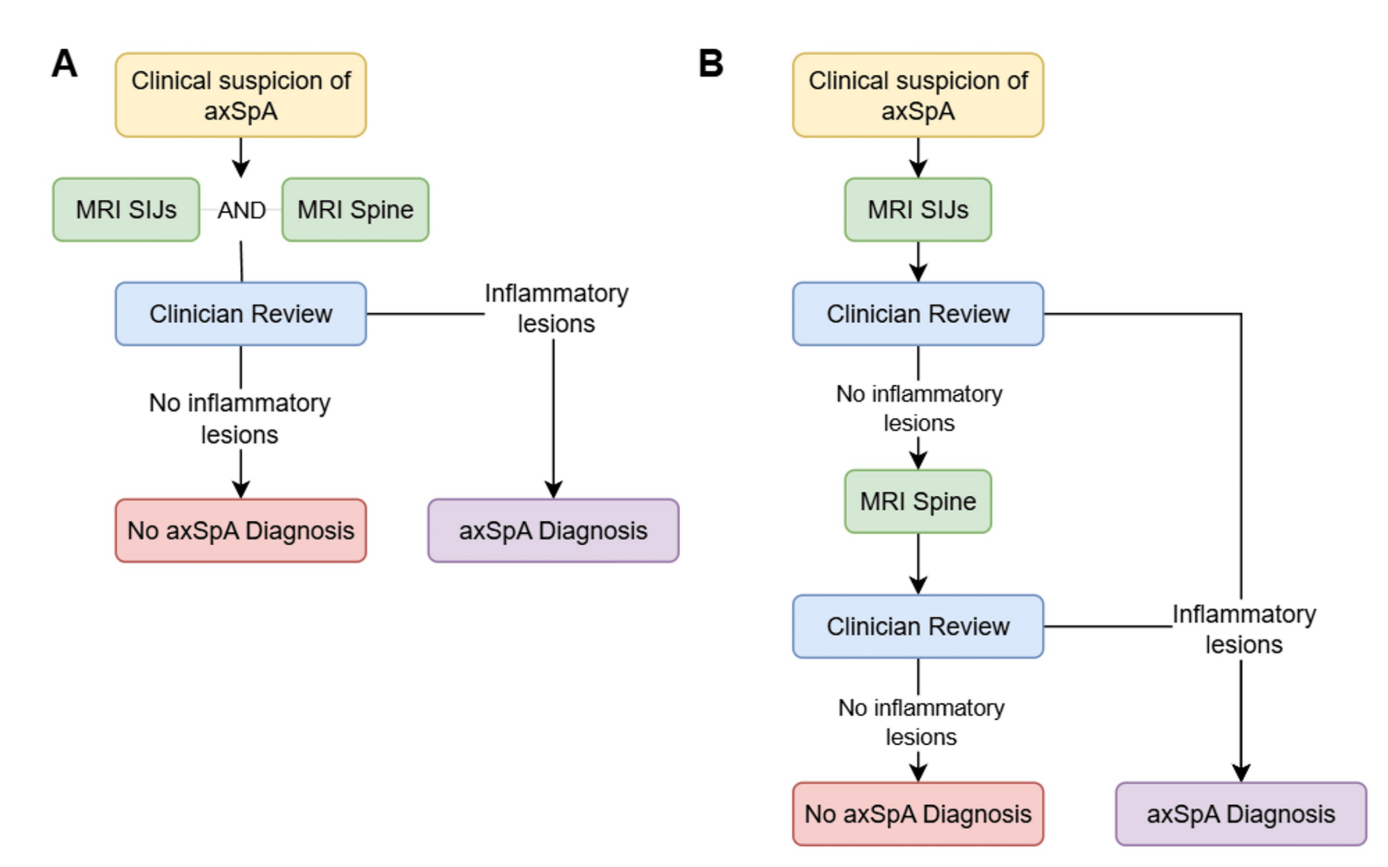
Proposed diagnostic imaging protocol. A: Current practice as recommended by BRITSpA guidance. B: Proposed sequential imaging of MRI SIJ, followed by MRI spine. axSpA: axial spondyloarthritis; MRI: magnetic resonance imaging; SIJ: sacroiliac joint; BRITSpA: British Society for Spondyloarthritis

We conducted a cost minimisation analysis, comparing two different diagnostic pathways that ultimately result in the equivalent diagnostic rate of axSpA. The first diagnostic pathway is our current practice, where all patients with suspected axSpA have an MRI of both SIJs and the whole spine. Our proposed diagnostic pathway involves sequential imaging; all patients would initially proceed with SIJ MRI, then this would be reviewed by a clinician and radiologist to determine whether the SIJ MRI would be sufficient to achieve a diagnosis of axSpA. If it is not sufficient, the patient proceeds with further MRI whole spine imaging. These diagnostic protocols are demonstrated in Figure 1.

We calculated resource usage in terms of MRI scanning time and cost per scan. MRI scanning time was provided by our local diagnostic imaging department as follows: MRI SIJ takes only 20 minutes of scanning time, and MRI whole spine takes 40 minutes of scanning time. Estimated costs of imaging were derived from the NHS National Cost Collection Data Publication: National Schedule 2023/24 [13]. Average unit cost for an MRI without contrast of one area (SIJs in this case) was £156, and an MRI without contrast of two or three areas (whole spine in this case) was £218. Based on our data collected, we calculated the total scanning time and cost savings by comparing both diagnostic pathways and calculate average savings per patient.

## Results

A total of 62 patients were included in our analysis. Mean age at diagnosis was 39.2 (±10.5) years, mean weight was 78.7 (±15.5) kg, and 32 (51.6%) patients were male. In terms of clinical characteristics, 19 patients (30.6%) were HLA-B27 positive, 11 patients (17.7%) had a concomitant diagnosis of psoriasis, eight patients (12.9%) had IBD, and seven patients (11.3%) had uveitis previously. The clinical and demographic characteristics are given in Table 1.

**Table 1:** Population demographics and clinical features (N=62) HLA: human leucocyte antigen; IBD: inflammatory bowel disease; MRI: magnetic resonance imaging; SIJ: sacroiliac joint

Characteristics	Value
Demographics	
Age at diagnosis (years), mean ± SD	39.2±10.5
Male, n (%)	32 (51.6%)
Weight (kg), mean ± SD	78.7±15.5
Clinical Features	
HLA-B27 positive, n (%)	19 (30.6%)
Psoriasis, n (%)	11 (17.7%)
IBD, n (%)	8 (12.9%)
Uveitis, n (%)	7 (11.3%)
Imaging features	
MRI SIJ with inflammatory lesions	51 (82.3%)
MRI SIJ with inflammatory lesions and MRI Spine without inflammatory lesions	35 (56.5%)
MRI Spine with inflammatory lesions	27 (43.5%)
MRI spine with inflammatory lesions and MRI SIJ without inflammatory lesions	11 (17.7%)
MRI spine with inflammatory lesions and MRI SIJ with inflammatory lesions	16 (25.8%)

Of the 62 patients, 51 demonstrated active inflammatory lesions diagnostic of axSpA as per the ASAS MRI working group’s diagnostic criteria on MRI SIJ. Only 11 patients (17.7%, 95%CI 13.1-32.0%) had non-diagnostic changes on MRI SIJ and so required MRI whole spine to demonstrate inflammatory lesions which met the ASAS diagnostic criteria for axSpA. As a result, 82.3% of patients would not have required an MRI of the whole spine in order to have radiological evidence of axSpA.

From a cost perspective, the current protocol of simultaneous MRI SIJ and spine imaging from this dataset would mean that all 62 patients in our cohort study would utilise 62 hours of MRI scanning time, costing £23,188. Our proposed diagnostic imaging protocol of sequential MRI SIJ followed by MRI spine would mean that all 62 patients would have MRI SIJ, but only 11 patients would proceed to have further MRI spine. Hence, the total MRI scanning time utilised by this protocol would be 28 hours, with a corresponding cost of £12,070. Hence, for this cohort of patients, our cost minimisation analysis demonstrates that by switching to a sequential scanning protocol, we would potentially save 34 hours of MRI scanning time and have cost savings of £11,118.

Within this cohort of 62 patients who were clinically felt to have a high likelihood of axSpA, 27 (43.5%) had changes on MRI whole spine which were diagnostic for axSpA, but only 11 (17.7%) of these had a non-diagnostic MRI SIJ. Therefore, by applying Bayes’ theorem and assuming a sensitivity of 82% for MRI SIJ in diagnosing axSpA as per our cohort, our data suggest that in such a high likelihood population, the probability of having a positive MRI whole spine despite a negative MRI SIJ is 42.9%.

Subsequently, we performed an exploratory logistical regression analysis to identify clinical and biochemical factors that may increase the likelihood of needing an MRI whole spine in addition to MRI SIJ (Table 2). We identified that patients with a history of IBD had an OR of 3.80 (95%CI 0.537-26.952, p = 0.181) of needing a spinal MRI in addition to SIJ MRI to achieve an axSpA diagnosis. In addition, age demonstrated an OR of 1.08 (95%CI 0.996-1.174, p = 0.061), nearing statistical significance. This suggests a trend that older patients possibly had an increased odds of needing an MRI spine in addition to MRI SIJ to achieve a diagnosis of axSpA. Other known factors, such as HLA-B27 status and history of psoriasis or uveitis, did not have a clinically significant impact on the likelihood of needing further spinal MRI to achieve a diagnosis of axSpA.

**Table 2:** Logistical regression analysis of predictors for need of spine MRI in addition to SIJ MRI A value of p<0.05 was considered statistically significant. HLA: human leucocyte antigen; SIJ: sacroiliac joint

Predictor Variable	Odds Ratio	95% Confidence Interval	P-value
Age	1.08	1.00 - 1.17	0.061
Weight	0.99	0.94 - 1.04	0.626
Male	2.73	0.54 - 13.80	0.223
HLA-B27 positive	1.29	0.28 - 6.02	0.748
Psoriasis	0.43	0.06 - 3.25	0.416
Inflammatory Bowel Disease	3.80	0.54 - 26.95	0.181

## Discussion

Our data show that a clinically significant proportion of patients needed only MRI SIJ to achieve a diagnosis of axSpA, but that there is a small proportion of patients who need both spinal and SIJ imaging to achieve an ASAS criteria diagnosis. This is reflective of previous studies, which have also demonstrated that a small proportion of patients with axSpA have inflammatory lesions only visible on spinal MRI, with a “negative” MRI SIJ [9,14-17]. However, it is debated in the literature whether spinal MRI combined with SIJ MRI adds diagnostic value in axSpA diagnosis [18]. Chan et al. (2020) [15] and Larbi et al. (2017) [14] identify 8-13% and 14.3% of patients with axSpA who have inflammatory lesions only on spinal MRI, which is similar to the results seen in our study. However, the SPACE (SPondyloArthritis Caught Early) and DESIR (Devenir des Spondylarthropathies Indifférenciées Récentes) cohorts, which are both longitudinal cohort studies of patients diagnosed with axSpA involving multiple centres, only report this for 1% and 2%, respectively [16]. This may be due to differences in the age of our cohort of patients, where, on average, patients within our study were diagnosed at an older age than patients within the SPACE and DESIR cohorts. The evidence from our study is supportive of the BRITSpA recommendations to image both spine and SIJ, as we clearly demonstrate that a small proportion of patients have only inflammatory lesions within the spine without any sacroiliitis.

To our knowledge, we are the first study to examine the resource impact of spinal MRI in combination with SIJ MRI. Our study demonstrates that 51 patients who had inflammatory lesions on MRI SIJ did not need to have a spinal MRI to achieve an axSpA diagnosis in this cohort; hence the MRI resources dedicated for these patients could have been utilised for other purposes. Consequently, we propose a system where all patients who need an MRI proceed to MRI scanning of SIJs first, followed by clinician and radiology review of imaging, before proceeding to further spinal MRI (Figure 1). This practice would combine the differing perspectives of the 2017 EULAR recommendation to only image SIJs and the BRITSpA-endorsed recommendations to image both SIJs and the whole spine, whilst optimising MRI resource usage [10,11].

Our Bayesian analysis has shown that in a patient with a high clinical suspicion of axSpA, there is a 42.9% chance that patients with an MRI SIJ that is negative for inflammatory lesions will have a positive MRI whole spine. Hence, the key message here is that MRI SIJ is useful to rule in a diagnosis of axSpA, but not enough to exclude a diagnosis of axSpA. This is in keeping with our current clinical practice at our tertiary centre and the recommendations from BRITSpA, highlighting the role of spinal MRI in the diagnosis of axSpA in patients who do not have sacroilitis demonstrated on MRI SIJ.

Finally, we note that our logistical regression analysis has demonstrated that patients with a history of concomitant IBD were more likely to need spinal MRI in addition to SIJs MRI to achieve a diagnosis of AxSpA. This clinically may have implications in the investigation for axSpA in this cohort of patients with IBD, potentially suggesting that these patients are more likely to have inflammatory lesions only in the vertebral column, meaning that MRI spine imaging is more likely to be needed in these patients. However, it is important to note that, given only a small number of our cohort had IBD, this would need to be validated in other centres and in a much larger cohort of axSpA patients.

Although the logistic regression analysis suggested a trend towards an increased likelihood of needing a spinal MRI in patients with IBD, this association did not reach statistical significance (p = 0.18). This may be due to the low numbers of patients with IBD in our cohort; hence, expanding this analysis to include larger numbers of patients with axSpA would be helpful in investigating this. More broadly, our overall sample size of 62 patients, while adequate for our primary endpoint, limits the statistical power of our exploratory analyses, as reflected in the wide confidence intervals around our effect estimates, as well as large p-values that do not reach statistical significance. Hence, further research with larger sample sizes is needed to confirm or refute this association. Further work investigating this should also investigate other clinical features that we have not examined here, such as family history, smoking, and symptom duration, as this may be helpful in identifying subpopulations of patients who are more likely to need MRI spine in combination with MRI SIJ.

Our cost-minimisation analysis focused on immediate imaging costs based on UK NHS tariffs and scanning time, without capturing potential downstream effects such as treatment delays, additional clinic visits, patient anxiety during the waiting period for sequential imaging, or long-term clinical outcomes. The cost estimates may not be directly applicable to other healthcare systems with different cost structures or reimbursement models. One other consideration with our suggested sequential imaging protocol in Figure 1 is that this may mean more clinician time is needed for clinicians to review the initial MRI SIJ result before deciding whether to proceed with further spinal MRI. This could potentially add to the delay in diagnosis with sequential imaging, contributing to patient anxiety.

Limitations

This study is subject to the limitations inherent in its retrospective design. The reliance on pre-existing radiology reports introduces the potential for variability in image acquisition and interpretation. This is particularly important to note as there can be significant inter-observer variation in MRI interpretation for axSpA. As discussed in our materials and methods section, we have attempted to limit the variability in interpretation by having any MRI imaging that was performed outside of our own tertiary centre re-reviewed by our specialist musculoskeletal radiologists within our weekly axSpA multidisciplinary team meeting. In addition, as mentioned above, our results from the logistical regression analysis are limited by the overall small sample size within our study and the fact that it is, by nature, a post-hoc exploratory analysis, represented by our wide confidence intervals and large p-values from our data. To investigate these trends further would require an appropriately large enough dataset, and it would be pertinent to include other clinical variables such as enthesitis, duration of symptoms, smoking, and family history of axSpA. Additionally, the single-centre design and specific referral criteria may limit the generalisability of our findings to other centres with differing patient populations or referral patterns. Note that our local referral criteria require patients to meet four out of the five criteria: age of onset of back pain less than 45 years-old, back pain that is gradual, improves with exercise, does not improve with rest, and pain at night which improves on getting up.

## Conclusions

Our study has demonstrated that most patients only needed MRI SIJ to achieve a diagnosis of axSpA and that there is a clinically significant proportion of patients who need further spinal MRI. We propose a sequential imaging protocol where all patients proceed initially with an MRI SIJ, with review of imaging before deciding whether to proceed with further MRI spine imaging, optimising the resource usage of MRI. Additionally, we have demonstrated that MRI SIJ is sufficient to rule in a diagnosis of axSpA, but that clinicians should consider further MRI whole spine imaging if the index of suspicion remains high despite the MRI SIJ demonstrating no active inflammatory lesions. Finally, our data has identified a possible trend that patients with IBD are more likely to have a negative MRI SIJ and hence require spinal imaging for diagnosis of axSpA, but this needs further investigation and validation in larger axSpA cohorts before application in clinical practice, as this was only demonstrated in a small, post-hoc exploratory analysis. 
